# P-1597. Severity of Omicron COVID-19 Variants: A Global Systematic Literature Review

**DOI:** 10.1093/ofid/ofaf695.1776

**Published:** 2026-01-11

**Authors:** Deepa Malhotra, Daniel Curcio, Moe H Kyaw, Rodrigo Sini de Almeida, Rajeev M Nepal, Pinelopi Nikolopoulou, Stephen Wiblin, Santiago M C Lopez, Irini Zografaki, Isabelle Whittle, Fiona Pearson, Sophie Pope, Fraser Williams

**Affiliations:** Pfizer, New York, NY; Pfizer Inc., Buenos Aires, Buenos Aires, Argentina; Pfizer, New York, NY; Pfizer, New York, NY; Pfizer Canada, Krikland, Quebec, Canada; Pfizer, New York, NY; Pfizer, New York, NY; Pfizer Inc, Collegeville, Pennsylvania; Pfizer, New York, NY; Adelphi Values PROVE, Manchester, England, United Kingdom; Adelphi Values PROVE, Manchester, England, United Kingdom; Adelphi Values PROVE, Manchester, England, United Kingdom; Adelphi Values PROVE, Manchester, England, United Kingdom

## Abstract

**Background:**

The continuous emergence of SARS-CoV-2 variants requires ongoing update to research into COVID-19 severity and clinical characteristics. This systematic review aimed to explore COVID-19 disease severity across variants from the Omicron dominance period onwards (i.e., BA.2, XBB, and JN.1 sublineages; see Figure 1).

**Table 1.**
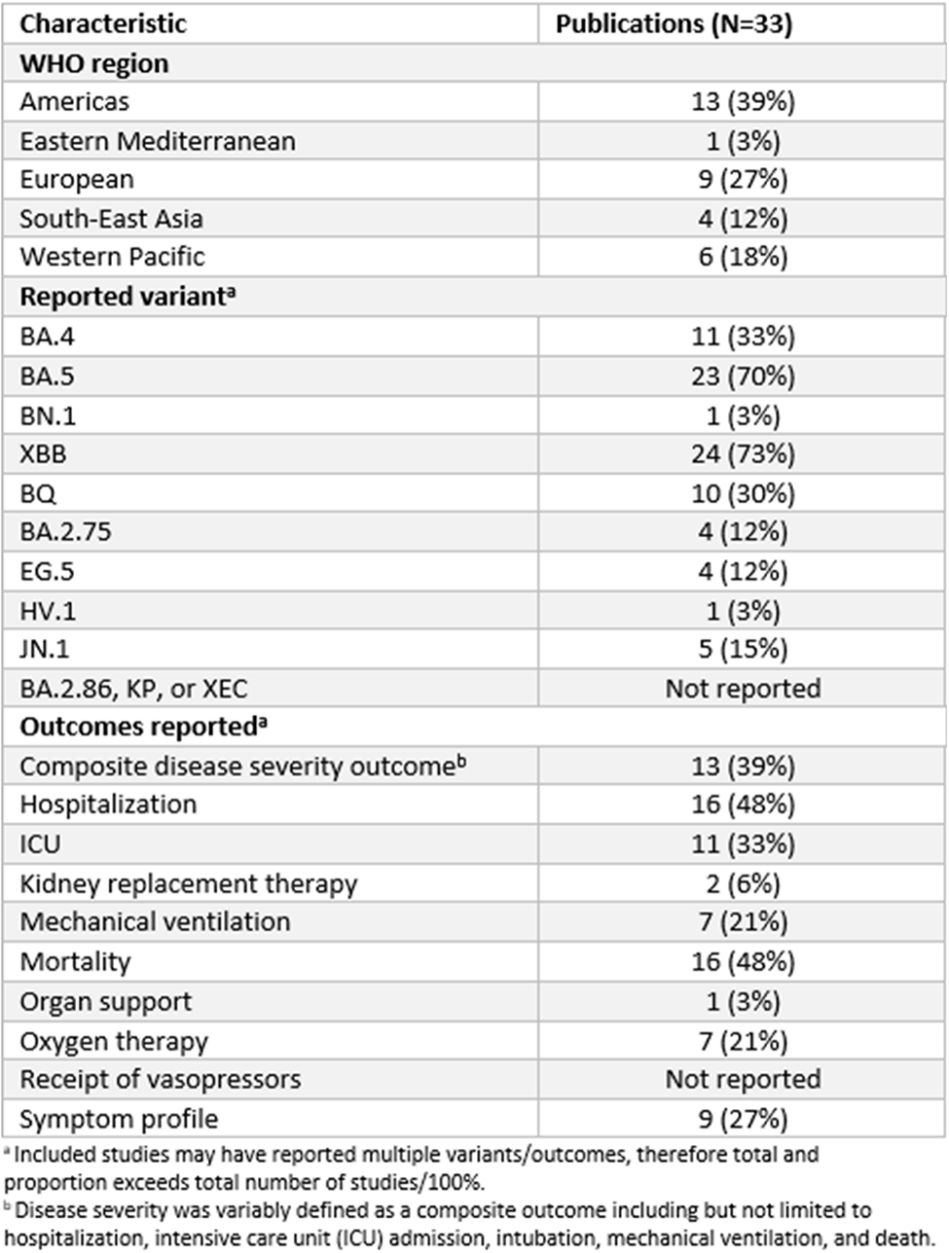
Summary of included publication study characteristics

**Figure 1.**
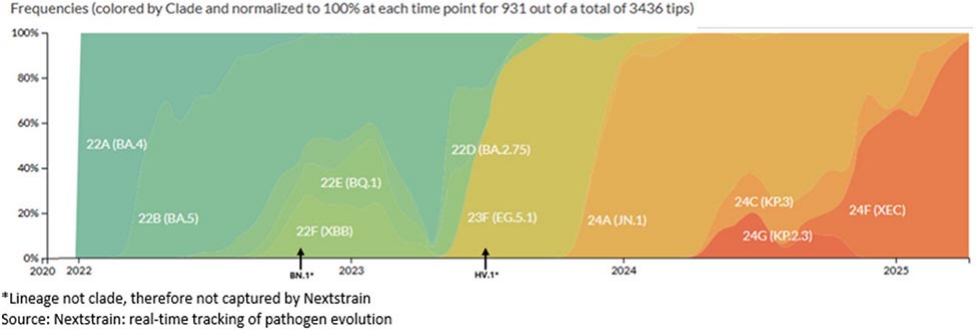
Global genomic epidemiology of the SARS-CoV-2 Omicron period from 2022 to current

**Methods:**

Systematic searches of Medline and Embase databases were conducted in November 2024 and supplemented by conference searches from 2022 to 2024 (PROSPERO ID: CRD42024619193). Eligible records compared acute COVID-19 outcomes for individuals testing positive for SARS-CoV-2 between variants, with variant determined through sequencing or study author definition of dominance period. Study quality was assessed using the Joanna Briggs Institute critical appraisal tools.

**Figure 2.**
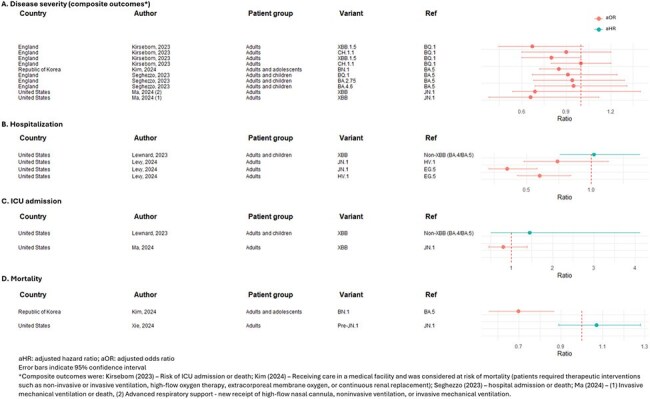
Direct comparison by variant period (all adjusted, not including subgroup analysis, estimate < 1 indicates increased severity during reference group period) of composite outcomes for disease severity (A), hospitalization (B), ICU admission (C), mortality (D)

**Figure:**
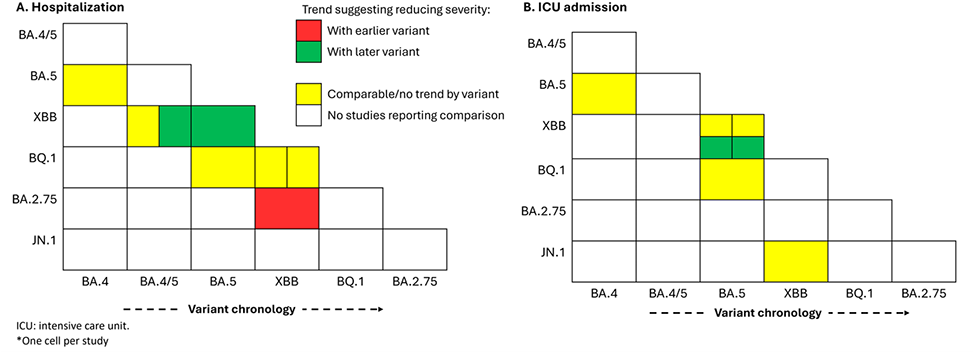
Heatmap summaries of included studies* reporting descriptive analyses without direct comparison between variants (not including subgroup analyses, outcomes reported for < 20 persons excluded) for hospitalization (A) and ICU admission (B)

**Results:**

Most studies (31/33; 94%) reported on earlier circulating Omicron variants (BA.4/5 and XBB; Table 1). Figure 2 summarizes studies that directly compared hospitalization, ICU admission, mortality, and composite measures of disease severity by SARS-CoV-2 variant. COVID-19 severity was comparable between variants across all outcomes, although two studies (6%) reported increased severity associated with earlier variants. A similar trend is depicted in Figure 3, which summarizes studies reporting descriptive analyses without direct comparison between variants (i.e., not included in Figure 2). The heatmaps depict analogous severity between BA.2 sublineages and most BA.2 versus XBB sublineage comparisons, although multiple studies indicated reduced severity with XBB infection versus BA.2 sublineages.

**Conclusion:**

The observed COVID-19 morbidity and mortality were comparable throughout Omicron sub-variants dominance. Comparison with JN.1 sublineages was limited by few reporting studies. The continued evolution of SARS-CoV-2 variants highlights the importance of continued vaccination campaigns with updated COVID-19 vaccines to better match circulating strains.

**Disclosures:**

Deepa Malhotra, MS MBA, Pfizer Inc.: Stocks/Bonds (Public Company) Daniel Curcio, M.Sc., Pfizer Inc.: Employee of Pfizer Inc. and may hold stock or stock options Moe H. Kyaw, PhD, Pfizer: Stocks/Bonds (Public Company) Rodrigo Sini de Almeida, Doctor, Pfizer: Board Member|Pfizer: Stocks/Bonds (Private Company) Rajeev M. Nepal, PhD, Pfizer Inc.: Employee of Pfizer Inc. and may hold stock or stock options Pinelopi Nikolopoulou, PhD, Pfizer: current employee|Pfizer: Stocks/Bonds (Private Company) Stephen Wiblin, Masters, Pfizer: Stocks/Bonds (Private Company) Santiago M.C. Lopez, MD, Pfizer Inc.: Employee of Pfizer Inc. and may hold stock or stock options|Pfizer Inc.: Stocks/Bonds (Public Company) Irini Zografaki, MPH, Pfizer: Current employee|Pfizer: Stocks/Bonds (Private Company) Isabelle Whittle, MSc, Pfizer Inc.: Contracted research support Fiona Pearson, PhD, Pfizer Inc. (Contracted research support): Advisor/Consultant Sophie Pope, BSc, Pfizer Inc.: Contracted research support Fraser Williams, MSc, Pfizer Inc.: Contracted research support

